# Characterizing the impact of pyrite addition on the efficiency of Fe^0^/H_2_O systems

**DOI:** 10.1038/s41598-021-81649-y

**Published:** 2021-01-27

**Authors:** Rui Hu, Xuesong Cui, Minhui Xiao, Willis Gwenzi, Chicgoua Noubactep

**Affiliations:** 1 School of Earth Science and Engineering, Hohai University, Fo Cheng Xi Road 10, Nanjing, 21180 China; 2 Department of Soil Science and Agricultural Engineering, Biosystems and Environmental Engineering Research Group, Faculty of Agriculture, University of Zimbabwe, P.O. Box MP207, Mount Pleasant, Harare Zimbabwe; 3 Department of Applied Geology, University of Göttingen, Goldschmidtstraße 3, 37077 Göttingen, Germany; 4 Centre for Modern Indian Studies (CeMIS), Universität Göttingen, Waldweg 26, 37073 Göttingen, Germany

**Keywords:** Environmental sciences, Chemistry, Materials science

## Abstract

The role of pyrite (FeS_2_) in the process of water treatment using metallic iron (Fe^0^) was investigated. FeS_2_ was used as a pH-shifting agent while methylene blue (MB) and methyl orange (MO) were used as an indicator of reactivity and model contaminant, respectively. The effect of the final pH value on the extent of MB discoloration was characterized using 5 g L^−1^ of a Fe^0^ specimen. pH variation was achieved by adding 0 to 30 g L^−1^ of FeS_2_. Quiescent batch experiments with Fe^0^/FeS_2_/sand systems (sand loading: 25 g L^−1^) and 20 mL of MB were performed for 41 days. Final pH values varied from 3.3 to 7.0. Results demonstrated that MB discoloration is only quantitative when the final pH value was larger than 4.5 and that adsorption and co-precipitation are the fundamental mechanisms of decontamination in Fe^0^/H_2_O systems. Such mechanisms are consistent with the effects of the pH value on the decontamination process.

## Introduction

The removal of anthropogenic and natural pollutants from aqueous systems is a major environmental concern^1–3^. Several technologies have been developed for water treatment over the past 170 years^1,4–8^. Technologies based on adsorption processes have been proven to be the most affordable and suitable for water treatment in low-income communities^1,5^. During the past three decades, metallic iron (Fe^0^) has been intensively used for in-situ environmental remediation^9–11^ and ex-situ water treatment^12–15^. However, controversy still exists on whether Fe^0^ acts as a reducing agent for several pollutants (e.g. selected chemicals)^11^ or a generator of contaminant scavengers (iron corrosion products–FeCPs) for all classes of pollutants (e.g. chemicals and pathogens)^16–18^. It is certain that, while undergoing oxidative dissolution, Fe^0^ induces contaminant removal in aqueous systems^19–23^, and this process can last for decades^24–27^. This ability to remove contaminants for prolonged periods has prompted the use of granular Fe^0^ for decentralized water treatment^12,14,28–30^.

Metallic iron (Fe^0^) and iron sulfide (FeS)-based materials (including pyrite–FeS_2_) are two important components of Fe^0^-based water treatment technology^11,22,31–33^. Both materials are reported to be stand-alone reducing agents that effectively degrade several aqueous contaminants^34,35^. In this context, Henderson and Demond^34,36^ have explicitly compared the suitability of both materials, and observed the superiority of FeS (including FeS_2_) over Fe^0^ with respect to the sustainability in terms of loss of permeability. During the past two decades, Fe^0^ and FeS_2_ have been often mixed in an effort to increase the efficiency of single-Fe^0^ systems^22,33,37–41^. However, FeS_2_ is mostly added to avoid the formation of a passive oxide scale (oxide film) which can hinder further reactions between the Fe^0^ and pollutants^41,42^. This application contradicts the successful use of FeS_2_ to improve the removal of non-reducible contaminants (e.g. As) in Fe^0^/H_2_O systems^22^. Thus, there is a need to understand the real mechanism by which FeS_2_ improves the efficiency of Fe^0^/H_2_O systems, irrespective of any redox transformation. The oxidative dissolution of both Fe^0^ (Eq. 1) and FeS_2_ (Eq. 2) typically releases Fe^2+^, which is also a stand-alone reducing agent for several contaminants^35^. Fe^2+^ from Eq. (1) and/or Eq. (2) can be further oxidized to Fe^3+^ (Eq. 3).

1 $$Fe^{0} + 2H^{+} \Rightarrow Fe^{2+}+H_{2}$$

2 $$FeS_{2} + 7/2 O_{2} + H_{2} O \Rightarrow Fe^{{2 + }} + 2SO_{4}^{{2-}} +2H^{+}$$

3 $$4Fe^{{2 + }}+O_{2} + 2H^{+} \Rightarrow 4Fe^{{3+}}+ 2HO^{-}$$

It is evident that both reactions depicted by Eqs. (1 and 3) consume acidity (H^+^), while reaction in Eq. 2 produces H^+^. According to the Le Chatelier’s principle, the reactions consuming H^+^ (Eq. 1 and 3) are accelerated by pyrite oxidation (Eq. 2). Here, the forward Fe^0^ dissolution is given priority as it is the main reactant, while less attention is paid to the possible inhibitory effect of FeS_2_ oxidation on Fe^0^ dissolution (production of Fe^2+^). In fact, using FeS_2_ to enhance iron corrosion is consistent with scientific principles (“Background to the experimental methodology”). However, this understanding provides no insights on the mechanisms of decontamination (adsorption, co-precipitation) and/or induced redox transformation of contaminants (degradation, precipitation). The stoichiometry of the reaction in Eq. (1) shows that 1 mol of Fe^0^ generates one mole of Fe^2+^ and one mole of H_2_, while 1 mol of FeS_2_ generates only one mole of Fe^2+^ (Eq. 2). Given that Fe^2+^ and H_2_ are stand-alone reducing agents, it is clear that there are more reducing agents (electron donors) in Fe^0^/H_2_O than in FeS_2_/H_2_O systems. The actual reductive characteristics of each system (i.e., Fe^0^/H_2_O, FeS_2_/H_2_O, and Fe^0^/FeS_2_/H_2_O) primarily depend on the relative dissolution kinetics of Fe^0^ and FeS_2_. According to their relative electrode potentials (-0.44 V for Fe^II^/Fe^0^ versus 0.25 V for S^III^/S^-I^), Fe^0^ should be transforming S^III^ species back to S^-I^ ones. This would correspond to the blocking of oxidative dissolution of Fe^0^. Although this reaction is thermodynamically feasible, it is masked by the more kinetically favourable FeS_2_ oxidation by dissolved O_2_. In the long-term (i.e., over the 41 d investigated herein), this process could contribute to the Fe^II^ cycle within a Fe^0^/FeS_2_ /H_2_O system.

The presentation above demonstrates the extreme complexity of the Fe^0^/FeS_2_/H_2_O and accounts for the controversies in the literature on the role of FeS_2_ in enhancing the efficiency of Fe^0^/H_2_O systems^22,33,41^. Thus, a detailed investigation of the efficiency of the Fe^0^/H_2_O system as influenced by the presence of FeS_2_ is warranted. A critical evaluation of the Fe^0^ literature suggests that one major limitation has been to test the efficiency of the Fe^0^/H_2_O system on individual contaminants or groups of contaminants (e.g. As, dyes, halogenated carbons)^22,28,33,41,43^. The net result is that increased adsorption, co-precipitation, degradation or precipitation have been reported as the supposed removal mechanisms. An innovative approach was introduced by Miyajima and colleagues using methylene blue (MB) as an indicator of reactivity for the Fe^0^/H_2_O system (MB method)^44,45^. The MB method exploits the differential adsorptive behaviour of MB onto sand and iron oxides or iron-coated sand^44–47^. Accordingly, in parallel experiments using constant amounts of sand and different Fe^0^ specimens, the most reactive system has been the one exhibiting the least MB discoloration, or the one producing the largest amount of iron oxides^47–49^. Note that the exact nature of the oxide is not very important, but its coating activity is the key aspect for the MB method. For example, Banerji and Chaudhari^12^ did not add any sand in their Fe^0^ bed to avoid oxide loss by sand coating. The objective of the current study was to characterize the impact of FeS_2_ addition on the reactivity of the Fe^0^/H_2_O system using the MB method. Thus, six different FeS_2_ mass loadings were used to achieve different final pH values (Eq. 2) (4.5 ≤ pHfinal ≤ 5.2).

## Material and methods

The present research is based on the chemistry of the Fe^0^/FeS_2_/sand/H_2_O system. Therefore, the operating mode of the system will be first discussed. In this study, various amounts of a reactive FeS_2_ mineral were added to a Fe^0^/sand mixture to investigate their effects on pH shifts and dye removal.

### Background to the experimental methodology

At neutral pH values, immersed reactive Fe^0^ corrodes and generates solid iron corrosion products (FeCPs), which progressively coat the surface of sand. The process of iron corrosion causes a pH shift to higher values (Eq. 1). The extent of sand coating depends among other factors on: (i) the Fe^0^ intrinsic reactivity, (ii) the volume of the solution, (iii) the initial pH value of the solution, (iv) the Fe^0^/sand ratio, and (v) the duration of the experiment. Under given experimental conditions, the removal efficiency of the system for individual contaminants depends on the final pH value, the extent of sand coating, and the availability of “free” FeCPs. The final pH value determines the speciation of the contaminant and the surface charges of sand and FeCPs^50^.

When a FeS_2_ mineral is added to a Fe^0^/sand system (at a given Fe^0^:sand ratio) a pH shift to lower values occurs. The extent of pH shift depends on the FeS_2_ intrinsic reactivity and the amounts added. Lower pH values avoid or delay sand coating and modify the speciation of dissolved contaminants. It then follows that, when FeS_2_ is added to a Fe^0^/sand mixture, there are two counteracting processes controlling the pH value of the system^37–39^. Previous results observed with the FeS_2_ mineral used in the current study^41^ suggest that pyrite dissolution occurs with much rapid kinetics than Fe^0^ corrosion. Consequently, the system will not achieve a steady state before the initial pH of the FeS_2_-free system is achieved. The larger the pH shift the larger the amount of FeCPs generated, which will in turn precipitate at pH > 4.5 and induce contaminant removal by adsorption and co-precipitation.

The methodology used for characterizing the impact of FeS_2_ on the efficiency of Fe^0^/H_2_O systems comprises monitoring the discoloration of a methylene blue solution (MB method) by Fe^0^/sand systems amended with various FeS_2_ amounts. Clearly, the availability of FeCPs and their reactivity is modified by lowering the initial pH value to various extents while observing MB discoloration in systems having a final pH value between 4.0 and 5.0. The discoloration of methyl orange (MO) in parallel experiments is used to support the findings based on the MB method. This approach is radically different from the conventional approach testing dyes as model contaminants^33,51^. For example, Chen et al.^33^ recently investigated the removal of three different azo dyes (Orange II, Reactive Red X-3B and Amido Black 10B) in the Fe^0^/FeS_2_/H_2_O system. All the three dyes are negatively charged, and were explicitly reported to be removed via reductive transformations. Following the conventional approach, Chen et al.^33^ monitored the concentrations of dyes, iron and protons (pH value), and performed solid phase characterizations using scanning electron microscopy (SEM), energy dispersive X-ray spectroscopy (EDS) and X-ray photoelectron spectroscopy (XPS). On the contrary, the MB method does not imply such solid phase characterizations because all FeCPs are positively charged^50^ and the extent to which they cover sand is reflected in the extent of MB discoloration.

### Solutions

#### Dyes

Methylene blue (MB) was used as a tracer of reactivity^47^, while methyl orange (MO) was a model organic contaminant^52^. Both dyes are widely used to characterize the suitability of various systems for water treatment^46,52–55^. The used dyes were of analytical grade. MB was supplied by Sinopharm Chemical Reagent Co. Ltd, Shanghai (China) and MO by Tianjin Chemical Reagent Research Institution Co. Ltd, Tianjin (China). The dyes were selected due to: (i) similarity in their molecular size, and (ii) differences in their affinity to positively charged iron oxides (Table 1)^55^. The initial dye concentration used was 10 mg L^−1^, equivalent to 31.5 μM for MB and 30.7 μM for MO. The working solutions were prepared by diluting concentrated stock solutions (3150 μM for MB and 3070 μM for MO) using deionized water. The pH values of the initial solutions were 6.5 (MB) and 7.0 (MO).

**Table 1:** Some physico-chemical characteristics of two tested dyes. MW stands for molecular weight.

Dye	Formula	MW (g mol^−1^)	Molecular seize (nm^3^)	Nature	_λmax_ (nm)
Methylene blue (MB)	C_16_H_18_ClN_3_S.3H_2_O	319.00	1.3 nm × 1.5 nm × 0.8 nm	Cationic	664.5
Methyl orange (MO)	C_14_H_12_N_3_O_3_NaS	327.34	1.19 nm × 0.67 nm × 0.38 nm	Anionic	464.0

#### Iron

A standard iron solution (1000 mg L^−1^) from General Research Institute for Nonferrous Metals was used to calibrate the UV/VIS spectrophotometer used for analysis. In preparation for spectrophotometric analysis, ascorbic acid was used to reduce Fe^III^ in solution to Fe^II^. 1,10 orthophenanthroline was used as reagent for Fe^II^ complexation^48,49,55^. Other chemicals used in this study included L( +)-ascorbic acid and L-ascorbic acid sodium salt. Ascorbic acid also degrades dyes (in particular MO) and eliminates interference during iron determination.

### Solid materials

#### Metallic iron (Fe^0^)

The Fe^0^ material was purchased from Shanghai Institute of Fine Technology (China). The material is available as scrap iron with a particle size between 0.05 and 5 mm. Its elemental composition as specified by the supplier was: Fe: > 99.99%; C: < 0.1%; N: < 0.1%; O: < 0.1%. Its k_Phen_ value is 13 mg h^−1^^56^. The k_Phen_ value is the kinetic constant of Fe^0^ dissolution in a 2 mM 1,10 orthophenanthroline solution, and characterizes the material’s intrinsic reactivity^57^. The material was used without any further pre-treatment. Fe^0^ was proven as a powerful discoloration agent for MB specifically because the discoloration agents are progressively generated in-situ^45,55^. Therefore, the discoloration capacity of the used Fe^0^ cannot be exhausted within the experimental duration used in the current study (41 d).

#### Sand

The sand conformed to the China ISO standard, and was used as received without any further pre-treatment or characterization. The particle size was between 1.25 and 2.00 mm. Sand was used because it is cheap and readily available and is widely used as admixing agent to prevent rapid permeability loss in Fe^0^/H_2_O systems^58^.

#### Pyrite (FeS_2_)

The FeS_2_ mineral was from Tongling City, Anhui province, China. The particle size was between 38 and 48 μm. Its weight composition was 46.0% Fe and 52.2% S, which is equivalent to a purity of 98.2%^41^. FeS_2_ was used because of its demonstrated suitability as a pH shifting agent in Fe^0^/H_2_O systems^37,41,59^.

### Dye discoloration experiments

Quiescent batch experiments were conducted in glass test tubes for an experimental duration of 41 d. Dye discoloration was initiated by adding 20.0 mL of the dye solution to a test tube containing 0.1 g of Fe^0^, 0.0 to 0.6 g of FeS_2_, 0.0 or 0.5 g of sand, and Fe^0^/FeS_2_/sand mixtures containing varying FeS_2_ loadings. Table 2 summarizes the aggregate content of the 8 Fe^0^/FeS_2_/sand systems and one operational reference (blank experiment), giving a total of 9 experimental treatments. Note that the pure Fe^0^ system (0.1 g of Fe^0^) is regarded as a Fe^0^/FeS_2_/sand system without FeS_2_ nor sand. The addition of sand was meant to avoid the compaction of the materials by gelatinous FeCPs via cementation^55^.

**Table 2:** Overview on the nine (9) investigated systems.

System	ZVI (g L^−1^)	Sand (g L^−1^)	Pyrite (g L^−1^)	Materials	Comments
Reference	0.0	0.0	0.0	None	Blank experiment
System 1	5.0	0.0	0.0	Fe^0^ alone	Blank for Fe^0^
System 2	0.0	25.0	0.0	Sand alone	Blank for sand
System 3	0.0	0.0	20.0	FeS_2_ alone	Blank for FeS_2_
System 5a	5.0	25.0	5.0	Fe^0^/sand/FeS_2_	First final pH value
System 5b	5.0	25.0	10.0	Fe^0^/sand/FeS_2_	Second final pH value
System 5c	5.0	25.0	20.0	Fe^0^/sand/FeS_2_	Third final pH value
System 5d	5.0	25.0	25.0	Fe^0^/sand/FeS_2_	Fourth final pH value
System 5e	5.0	25.0	30.0	Fe^0^/sand/FeS_2_	Fifth final pH value

The efficiency of individual Fe^0^ systems for dye discoloration was characterized at laboratory temperature (about 25 ± 2 °C). The final pH value, the iron concentrations and the residual dye concentrations were recorded. All experiments were carried out in triplicates under laboratory (oxic) conditions. The test tubes were protected from direct sunlight.

### Analytical methods

Aqueous dye and iron concentrations were determined by a 752 UV/VIS spectrophotometer (automatic) (Shanghai Jing Hua Technology Instrument Co. LTD). The working wavelengths for MB, MO and iron were 664.5, 464.0 and 510.0 nm, respectively. Cuvettes with a 1.0 cm light path were used. The iron determination followed the 1,10 orthophenanthroline method^60^. The spectrophotometer was calibrated for dye concentrations ≤ 15.0 mg L^−1^ and iron concentration ≤ 10.0 mg L^−1^. The pH value was measured by combined glass electrodes (INESA Scientific Instrument Co. China).

### Presentation of experimental results

In order to characterize the magnitude of the tested systems for dye discoloration, the discoloration efficiency (E) was calculated (Eq. (4)). After the determination of the residual dye concentration (C_t_), the corresponding percent dye discoloration efficiency (E value) was calculated as:

4 $$E = [1 - (Ct/C0)] \times 100\%$$

where C_0_ is the initial aqueous dye concentration (about 10.0 mg L^−1^), while C_t_ gives the final dye concentration at sampling time (t). The operational initial concentration (C_0_) for each case was acquired from a triplicate control experiment without additive materials (blank). This procedure was mainly meant to account for experimental errors due to dye adsorption onto the walls of the test tubes.

## Results and discussion

### Dye discoloration in single-aggregate and ternary-aggregate systems

**Figure 1:**
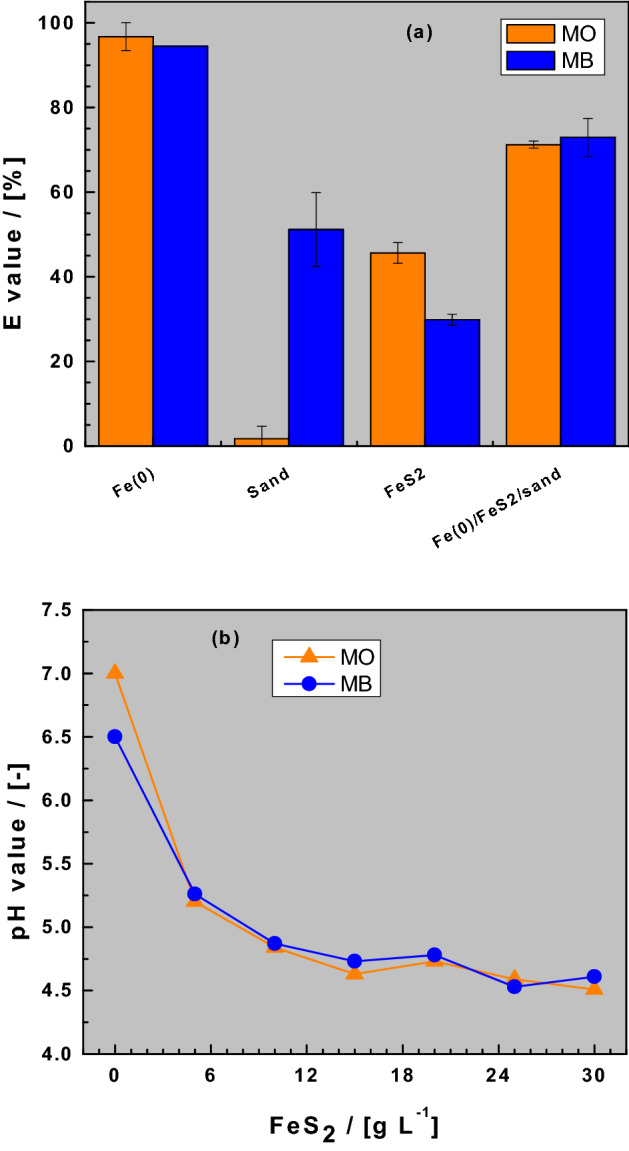
Changes of the dye discoloration efficiency (E values) in single-aggregate and ternary system (**a**) and changes of final pH value as a function of the FeS_2_ dose (**b**). Experimental conditions: V = 20 mL, m_iron_ = 0.0 or 0.1 g, m_sand_ = 0.0 or 0.5 g, m_pyrite_ 0 to 0.6 g, and t = 41 d. The lines are not fitting functions, they simply connect points to facilitate visualization.

Figure 1a compares the extent of dye discoloration in the four investigated systems: (i) single-Fe^0^, (ii) single-FeS_2_, (iii) single-sand, and (iv) Fe^0^/FeS_2_/sand. Figure 1b shows the final pH variation with varying FeS_2_ doses in the ternary Fe^0^/FeS_2_/sand systems. Figure 1a clearly shows that there was no MO discoloration in the pure sand system (E = 0%). The E values for both dyes in all other systems were larger than 30%. The uniqueness of the single-sand system (100% sand) relative to the other three systems is that it contains no in-situ generated FeCPs (Table 3). Therefore, only sand with its negatively charged surface^46,50^ is available for dye discoloration via pure electrostatic interactions. Strong surface interactions with positively charged species is thus responsible for the observed MB discoloration, but no MO discoloration occurs in the single-sand system^44,61^. As quiescent batch experiments were performed (no advection), diffusive mass transfer in the bulk solution and/or in the pores of generated FeCPs are the rate-limiting steps for the discoloration process.

**Table 3:** Time-dependent inventory of reactive species in the four investigated systems. t_0_ corresponds to the start of the experiment, while t_∞_ corresponds to Fe^0^ depletion.

System	Fe^0^	FeS_2_	Sand	Fe^0^/FeS_2_/Sand
t_0_ = 0	Fe^0^	FeS_2_	Sand	Fe^0^ + FeS_2_ + Sand
t > t_o_	Fe^0^ + FeCPs	FeS_2_ + FeCPs	Sand	Fe^0^ + FeS_2_ + Sand + FeCPs
t_∞_	FeCPs	FeS_2_ + FeCPs	Sand	FeS_2_ + Sand + FeCPs

It is assumed that because of Fe^II^ cycling FeS_2_ will be depleted the last. FeCPs = Fe corrosion products. FeCPs can be free or coated on sand.

The absence of MO discoloration in the single-sand system is the most important observation from these experiments. MO discoloration is observed in all other systems and is consistently more intensive than MB discoloration in single-Fe^0^ and single-FeS_2_ systems (“Dye discoloration in Fe^0^/sand/H_2_O systems”). For the Fe^0^/FeS_2_/sand system there is no pronounced difference in the discoloration of both dyes. It is recalled that the Fe^0^/FeS_2_/sand system contains 5 g L^−1^ Fe^0^, 25 g L^−1^ of sand and 20 g L^−1^ of FeS_2_. Thus, according to Table 2, the extent of dye discoloration depends on: (i) the availability of adsorption sites on inert sand (adsorption on sand), (ii) the extent to which sand is covered by in-situ generated FeCPs (selective adsorption on sand and/or FeCPs), and (iii) the extent to which excess FeCPs can be freely precipitated in the bulk solution (co-precipitation with FeCPs) (see Table 3). In this case, free precipitation herein means FeCPs that are not coating the sand surface (“Background to the experimental methodology”)^37,38,44,61^.

The merit of the experimental design is to demonstrate dye discoloration in a Fe^0^/sand/H_2_O systems as the FeS_2_ mass loadings vary from 0 to 30 g L^−1^. Figure 1b clearly shows that varying the FeS_2_ mass loading under the experimental conditions has resulted in various final pH values, ranging from 4.5 to 7.0 (Table 4). The results summarized in Table 4 also show lower pH values in all FeS_2_-containing systems with the single-FeS_2_ having a pH value of 3.3 ± 0.2 for both dyes. Notably, comparison of MO versus MB showed no profound difference in the final pH value (4.7 vs 4.8), the iron concentration (63 vs 65) and the E values (71 vs 73) in the ternary system with a FeS_2_ mass loading of 20 g L^−1^. An exception was the Fe^0^/H_2_O system without FeS_2_ (i.e., [FeS_2_] = 0 g L^−1^) for which a significant difference in the pH value was observed (6.5 for MB vs. 7.0 for MO). This corresponds to the pH value of the respective initial dye solutions (“Solutions”). The ternary-aggregate system used in this case comprised of 20 g L^−1^ FeS_2_. For a better illustration of the role of the FeS_2_ mineral in the process of dye discoloration in Fe^0^/H_2_O systems, three lower (5, 12 and 18 g L^−1^) and two higher (24 and 30 g L^−1^) FeS_2_ doses were also used.

**Table 4:** Variations of the pH value, the iron concentration ([Fe]) and the extent of dye discoloration (E) in the single-aggregate and the ternary systems after 41 days of equilibration.

System	pH (–)	[Fe] (mg L^−1^)	E (%)	pH (–)	[Fe] (mg L^−1^)	E (%)
		MO			MB	
Reference	6.9 ± 0.1	0.0 ± 0.0	0.0 ± 1.5	6.7 ± 0.1	0.0 ± 0.0	0.0 ± 1.5
Fe^0^	7.0 ± 0.2	0.7 ± 0.1	96.8 ± 3.3	6.5 ± 0.3	0.6 ± 0.2	94.5 ± 2.5
Sand	6.9 ± 0.1	0.0 ± 0.4	1.7 ± 3.0	6.7 ± 0.2	0.0 ± 0.9	51.2 ± 8.7
FeS_2_	3.3 ± 0.1	72.3 ± 4.8	45.6 ± 2.4	3.4 ± 0.1	68.0 ± 4.2	29.8 ± 1.3
Fe^0^/sand/FeS_2_	4.7 ± 0.1	62.9 ± 4.4	71.2 ± 0.8	4.8 ± 0.1	65.4 ± 3.4	72.9 ± 4.5

### Iron release in Fe^0^/sand/H_2_O systems

**Figure 2:**
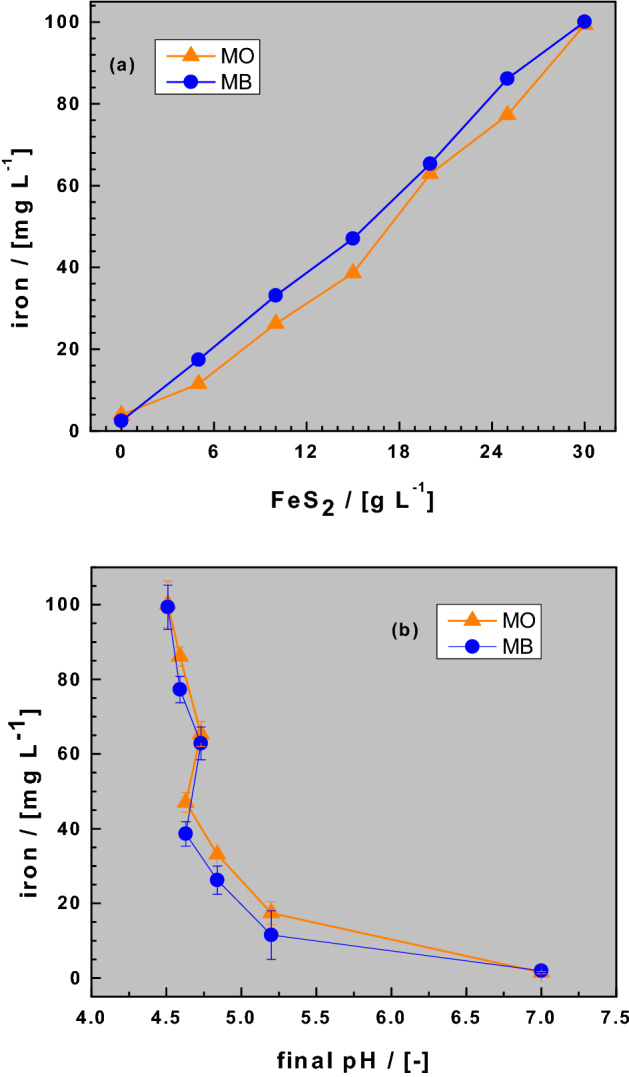
Changes of the iron concentration as function of the pyrite dose (**a**) and the final pH value (**b**). Experimental conditions: V = 20 mL, m_iron_ = 0.1 g, m_sand_ = 0.5 g, m_pyrite_ 0 to 0.6 g, and t = 41 d. The lines are not fitting functions, they simply connect points to facilitate visualization.

Figure 2 compares the iron concentration in the Fe^0^/sand/H_2_O systems as the FeS_2_ loadings vary from 0 to 30 g L^−1^. Figure 1b and Table 4 have shown a monotonous, but non-linear decrease of the pH value with increasing FeS_2_ mass loadings. Contrary, Fig. 2a shows a monotonous and linear increase of the iron concentration with increasing FeS_2_ mass loadings. For a constant FeS_2_ loading, the final pH values (Fig. 1b) and the [Fe] values (Fig. 2a) were almost the same for both dyes. This observation suggests that the differential behavior of MB and MO in interacting with the involved aggregates, particularly Fe^0^ and sand, is not reflected in changes of the pH value. Thus, the final pH value arises from the pseudo-steady state equilibrium between two antagonistic processes: (i) Fe^0^ corrosion which increases the pH value (Eq. 1), and (ii) FeS_2_ dissolution which decreases the pH value (“Background to the experimental methodology”). Figure 2b clearly shows that lower pH values correspond to higher [Fe] values. Specifically, the [Fe] values dropped from 100 mg L^−1^ for pH 4.7 to 0.7 mg L^−1^ for pH 7.0. A sharp decrease of the iron concentration between pH 4.7 and 5.5 is observed and corresponds to the solubility behavior of Fe under oxic conditions^62,63^. Again, there is no significant difference evident between both dyes.

The fact that iron dissolution from any reactive material increases with decreasing pH value is intuitive (Fig. 2a). However, the extent to which iron is dissolved under any given operational conditions should be characterized (Fig. 2b), and their impact on the investigated process (dye discoloration in this case) discussed. Past efforts to characterize the Fe^0^/FeS_2_ system have not properly considered these issues as the final pH value was not always recorded and/or not used in discussing the results^22,41^. Moreover, in earlier efforts it was commonplace to vary both the initial pH value and the FeS_2_ loading (Table 5), thereby making it difficult to determine the effect of each parameter. By using only various FeS_2_ loadings to shift the pH value of a Fe^0^/sand/H_2_O system, the present study is an extension of earlier efforts from the early 2000s^37–39^. Moreover, the current study applied a recently developed tool using MB as an indicator of Fe^0^ reactivity^44,45,47^.

**Table 5:** Overview of selected experimental conditions used for batch experiments in investigating the role of FeS_2_ in enhancing the efficiency of Fe^0^/H_2_O systems.

X	t	pH_0_	Fe^0^	FeS_2_	FeS_2_/Fe^0^	V	Stirring	References
–	(h or d)	(–)	(g L^−1^)	(g L^−1^)	(–)	(mL)	(rpm)	
CT ^(1)^	1 h	6.5 to 12.4	5	5.0 to 30.0	1	25	170	^59^
U	120 d	7.2	5	15	3	22	0	^37–39^
As	3 h	3.0 to 9.0	n.s	n.s	0.3 to 1.7	500	400	^22^
NB ^(2)^	5 h	5.0 to 10.0	0.5	0.5 to 3.0	1.0 to 6.0	150	200	^41^
MB & MO	41 h	7.0	5.0	5.0 to 30.0	1.0 to 6.0	20	0	TR

X stands for the used contaminant. Only this research and refs.^37–39^ have used quiescent systems and experimental durations longer that one day.

^(1)^ Carbon tetrachloride; ^(2)^ Nitrobenzene; *TR* This research.

### Dye discoloration in Fe^0^/sand/H_2_O systems

**Figure 3:**
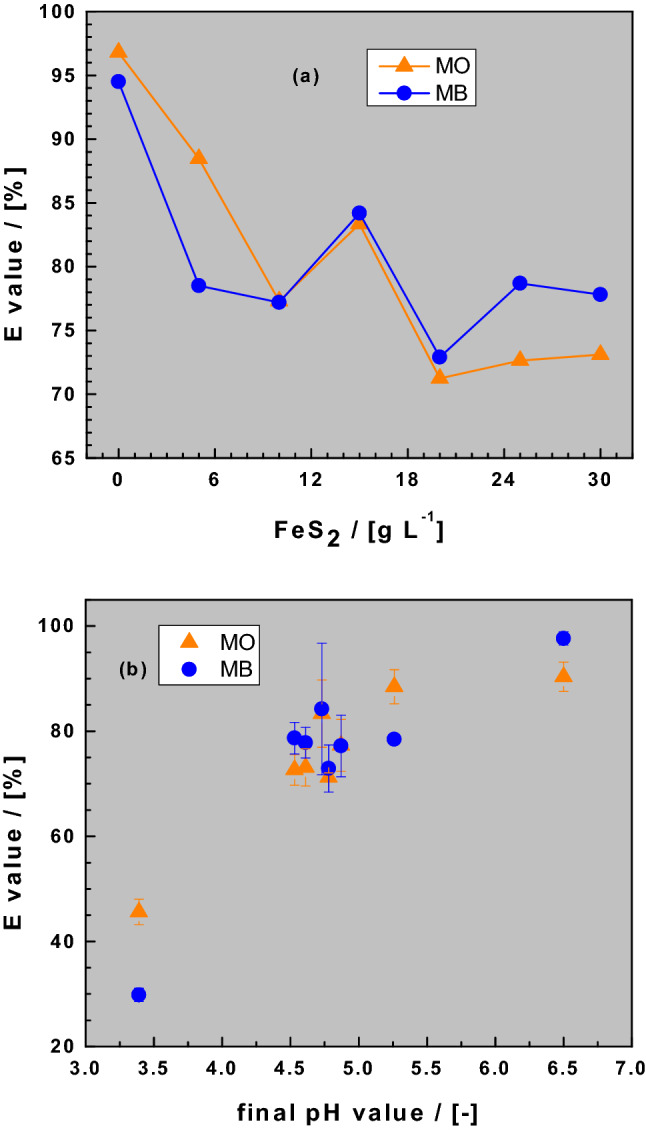
Changes of the dye discoloration efficiency (E values) as function of the pyrite dose (**a**) and the final pH value (**b**). Experimental conditions: V = 20 mL, m_iron_ = 0.1 g, m_sand_ = 0.5 g, m_pyrite_ 0 to 0.6 g, and t = 41 d. The lines are not fitting functions, they simply connect points to facilitate visualization.

Figure 3a shows the extent of dye discoloration (E values) by the ternary system as the FeS_2_ loadings increase from 0 to 30 g L^−1^. Figure 3b depicts the variation of E values as a function of the final pH value. It is evident that there is a general linear decrease in E value with increasing FeS_2_ loading or decreasing pH values (Fig. 2a). However, three important issues have to be considered: (i) the highest E value for each system corresponds to [FeS_2_] = 0 g L^−1^ (Issue 1); (ii) for [FeS_2_] = 5 g L^−1^ and [FeS_2_] > 20 g L^−1^, there is a significant difference in the E values for both dyes (Issue 2), and (iii) for [FeS_2_] = 10, 15 and 20 g L^−1^, there is no profound difference in the E values for both dyes (Issue 3). It is evident that such results would be considered controversial if coming from different independent studies. That is why considering the experimental conditions is crucial in discussing experimental result from independent studies^18,23^. For the so-called bottle-point technique used herein^64^, relevant operational variables include: Fe^0^ pre-treatment, Fe^0^ particle size (mm, mm, nm), Fe^0^ mass loading, volume of used vessels, volume of solution, buffer application, mixing type (e.g. stirring, shaking), mixing intensities (e.g. 200 rpm), and experimental duration^43^. A more detailed discussion of the three issues is given below.

Issue 1 implies that FeS_2_ addition inhibits the efficiency of Fe^0^/H_2_O systems for dye discoloration. Similar results were reported by Noubactep et al.^37–39^ while investigating U^VI^ removal in Fe^0^/H_2_O systems. Note that the single-FeS_2_S_2_ discolored both dyes (Fig. 1a). This observation raises questions about the assertion that FeS_2_ increases contaminant removal in Fe^0^/H_2_O systems^22,33,41^.

Issue 2 can be regarded as a striking feature as there is either a larger extent of MO discoloration relative to that of MB ([FeS_2_] = 5 g L^−1^) or the opposite ([FeS_2_] > 20 g L^−1^). Note that the ion-selectivity principle of the Fe^0^/H_2_O system implies that in the presence of FeCPs in aqueous systems, the anionic MO is better discolored than the cationic MB. This is obviously the case at [FeS_2_] = 5 g L^−1^ where enough FeCPs is generated to cover the surface of sand, thereby inducing a larger E value for MO than for MB. The observation that for Fe^0^/H_2_O systems without FeS_2_ (i.e., at [FeS_2_] = 0 g L^−1^), MO discoloration was only slightly higher than that of MB is also noteworthy. This indicates that for the experimental duration used in the current study (41 d), enough FeCPs were generated to co-precipitate both dyes. For further clarification of this issue, a binary Fe^0^/sand system should have been investigated, but this was beyond the scope of the current study. The higher MB discoloration relative to MO observed at [FeS_2_] > 20 g L^−1^ is explained by the formation of complexes between Fe and MO which delay co-precipitation^55,61,65^.

Finally, Issue 3 can also be regarded as a striking observation because despite all differences (solubility, affinity), there is no difference in the E values for the two dyes. It can be assumed that, under the experimental conditions, MB and MO which have almost the same molecular size (Table 1) are both discolored by co-precipitation^66^. This assumption is corroborated by results in Fig. 3b showing clearly that there is no quantitative dye discoloration (E > 60%) at pH < 4.5. This corresponds to the observations of Noubactep et al.^37–39^ for the Fe^0^/U^VI^/H_2_O system. The fact that MB, MO and U^VI^ exhibited very similar behaviors in the Fe^0^/FeS_2_/H_2_O system is an indication that contaminant removal might be a pure positive side effect of aqueous iron corrosion. The most tangible proof for this assertion is the kinetics of Fe^2+^ oxidation by dissolved oxygen (O_2_). According to Langmuir^67^, the kinetics of this reaction increases by a factor 65 between pH 4.0 and 5.0. Thus, quantitative dye discoloration is observed only in systems where Fe^2+^ oxidation to Fe^3+^ was quantitative for the 41-d experimental period. The in-situ generated Fe^III^ precipitates are good contaminant scavengers.

### Mechanisms of contaminant removal in Fe^0^/H_2_O systems

This study has investigated the effect of FeS_2_ addition on the efficiency of Fe^0^/sand systems for MB and MO discoloration. No enhanced dye discoloration could be attributed to FeS_2_ addition at mass loading of 0 to 30 g L^−1^ for 41 d. Two questions arise: First, why is there no increased dye discoloration in a context where the expected pH shift and increased iron dissolution are evident? (Question 1). It is noteworthy that each individual aggregate (Fe^0^, FeS_2_, sand) tested herein can achieve MB discoloration as depicted in Fig. 1a. Second, why did the ternary system perform far lower that single- Fe^0^ systems? (Question 2). By applying a known experimental approach consisting of varying individual operational parameters to better understand complex systems^17,68–70^, and accounting for the relative slow kinetics of Fe^0^ and FeS_2_ dissolution^37–39^, this study has adopted a novel approach to answer Questions 1 and 2. Specifically, the current study assessed the role of FeS_2_ in enhancing contaminant removal in Fe^0^/H_2_O system. MB is used herein as an operational reactivity tracer (“Introduction”) and the achieved results corroborated earlier reports on U(VI) removal^37–39^, and account for discrepancies and inconsistencies reported in literature^33,41,49,70^.

The evidence that FeS_2_ oxidation produces acidity (Eq. 2) is corroborated in the current study (Fig. 2a). By consuming acidity, Fe^0^ (Eq. 1) and Fe^2+^ (Eq. 3) oxidation are accelerated by Eq. (2) (Le Chatelier’s principle). Fe^3+^ from Eq. (2) catalyses FeS_2_ oxidation and produced less soluble Fe(OH)_3_. Thus, mixing Fe^0^ and FeS_2_ can be regarded as continuously generating less soluble Fe(OH)_3_, until one of the reactants is depleted or until a pseudo-steady state is established. This work posits that Fe(OH)_3_ discolors the dye solutions mainly by co-precipitation^66,70^. Thus, dye discoloration is only quantitative when Fe(OH)_3_ precipitation is intensive (pH > 4.5). Having used quiescent systems, various final pH values could be achieved, thereby confirming the pH shifting function of FeS_2_^37,41,59^. However, the extent of dye discoloration depends on the amount of free in-situ generated Fe(OH)_3_^55,70^ which is determined by the kinetics of Fe^2+^ oxidation by dissolved O_2_^67^. As expected, for a longer experimental duration (t > 41 d), the efficiency of the ternary mixture will surpass that of the single- Fe^0^ systems^37–39,70^. This answers Question 1, and demonstrates that enhanced dye discoloration needs more time to occur under quiescent conditions^37–39^. Accordingly, the documented delay of quantitative dye discoloration is not a negation of the view that FeS_2_ addition enhances the efficiency of Fe^0^/H_2_O system^33,40,41,70^. This study aims to better understand why Fe^0^/H_2_O systems are more efficient upon the addition of pyrite (FeS_2_) relative to those without pyrite.

In a ternary Fe^0^/FeS_2_/sand system, sand is non-reactive (inert) and is in-situ coated by iron oxides from the dissolution of the two other aggregates (“Background to the experimental methodology” and Table 2). This in-situ coating of sand delays the availability of free Fe(OH)_3_ for dye co-precipitation. Initially, MB and Fe^2+^/Fe^3+^ compete for adsorptive removal at the negatively charged sand surface^70,71^. Once the sand surface is completely coated, it will be no longer attractive for MB. This competition for active adsorption sites explains the observations in Fig. 3a. Note that neither Fe^0^ nor FeS_2_ are the discoloring agents, but rather the products of their oxidative dissolution which are variably available in the investigated systems (Table 3). To completely answer Question 2, the ternary mixture performed less than the single-aggregate systems because: (i) sand is in-situ coated, thereby retarding the availability of free Fe(OH)_3_, and (ii) the synergy of Fe^0^ and FeS_2_ has not yet produced enough free Fe(OH)_3_. The latter is the case whenever the pH value of the system has not exceeded 4.5 (Fig. 3b).

The presentation until this point has not addressed the redox properties of MB and MO. The thermodynamics predict MO reduction by Fe^0^^55,61^. The results reported herein demonstrate that even the ion-selective nature of the individual dyes was not the key factor accounting for dye discoloration when the pH was lower than 4.5. Thus, regardless of any redox properties, the current work has demonstrated that Fe^0^-based systems are only efficient when the final pH value is larger than 4.5. Unlike the current study, several previous works have mostly failed to record the final pH values of their systems and use them in their discussion (Table 5).

### Significance of the results

Fe^0^-based systems have been important components of the water treatment industry for the past 170 years^8,27,70^. Research reported before 1990 is not really considered by current active scientists whose starting point is the advent of in-situ permeable reactive barriers (PRBs), and the premise that Fe^0^ is an environmental reducing agent^18,23,72,73^. Conventional PRBs use micro-scale or granular Fe^0^ specimens (g Fe^0^). During the past two decades, some tools have been developed to improve the efficiency of g Fe^0^. In this regard, the following three tools have been introduced: (i) using nano-scale Fe^0^, (ii) alloying g Fe^0^ with metals such as Pd or Ni (also at nano-scale), and (iii) admixing another aggregate with g Fe^0^^11,24,25^. The Fe^0^/FeS_2_/sand system investigated herein is part of the third category. It has been reported that in sulfide-containing environments, using g Fe^0^ results in the formation of iron sulfides which are conductive and sustain electron transfer from Fe^0^ to the contaminant^22,74–76^. On the other hand, such iron sulfides are stand-alone reducing agents for the reductive transformations of many contaminants^70,77,78^. Because Fe^0^ and FeS_2_ have in common the release of Fe^II^ species, it can be assumed that the material containing more Fe will be first passivated by Fe^III^ species. However, when both materials are mixed, FeS_2_ accelerates Fe^0^ corrosion and none of both materials is really available for quantitative reductive transformation of other foreign species, including contaminants. Consequently, any observed enhancement of contaminant removal in a Fe^0^/H_2_O system by virtue of the presence of FeS_2_ is an indirect process. This assertion was elegantly demonstrated in the present study by slowing down the process of iron precipitation via addition of various FeS_2_ doses to the same Fe^0^/sand system for 41 d. It then follows that, FeS_2_ is mostly a pH shifting agent for the Fe^0^/H_2_O system^37,59,70^.

**Figure 4:**
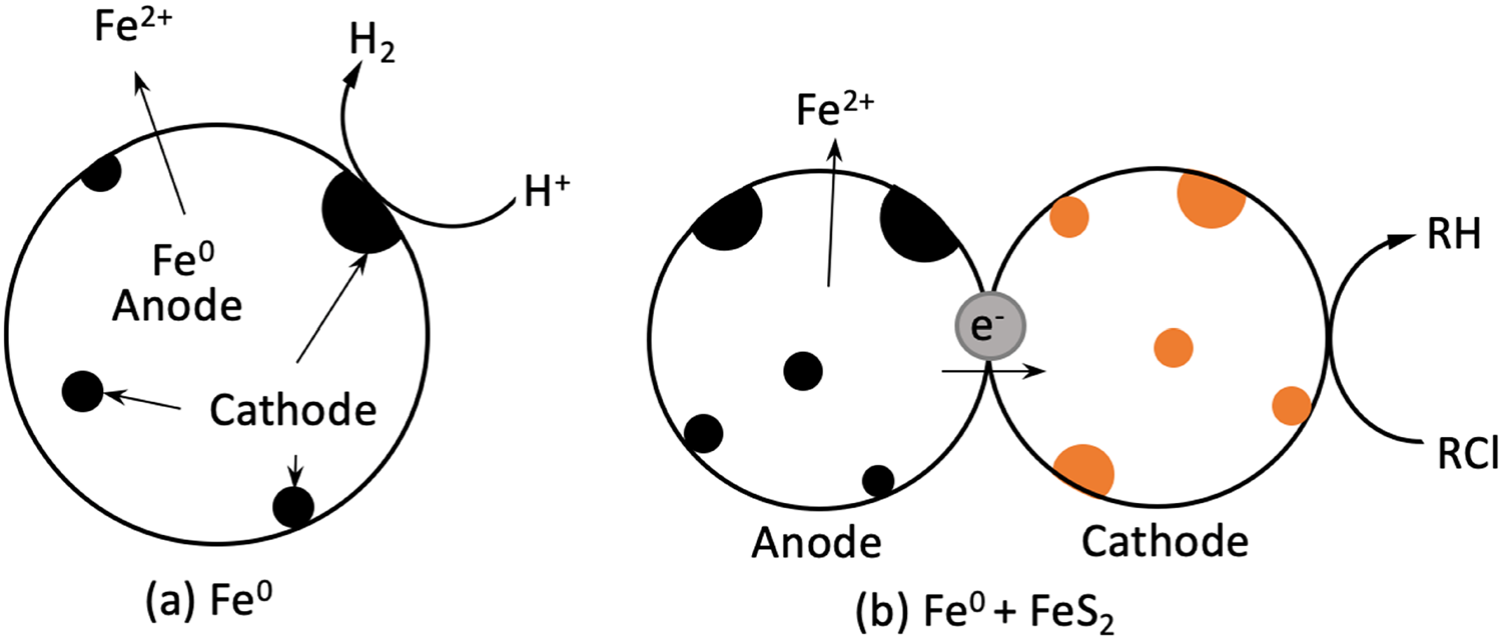
Schematic diagram of interactions between metallic iron (Fe^0^), pyrite (FeS_2_) and contaminants (RCl) in the remediation process: (**a**) Fe^0^/H_2_O and (**b**) Fe^0^/FeS_2_/H_2_O. In the Fe^0^/H_2_O system, anode and cathode are different sites on the same grain. In the Fe^0^/FeS_2_/H_2_O system, granular Fe^0^ is additionally the anode and FeS_2_ the cathode. The representation is based on the knowledge that Fe^0^ is not a reducing agent. Therefore, electrochemical contaminant reduction of RCl is possible in (**a**) and not in (**b**).

Table 5 reveals that all other investigations on the Fe^0^/FeS_2_ system were performed under shaken/stirred conditions. However, under such conditions, the target FeS_2_ intrinsic properties (including semi-conduction) are undermined. For example, how can FeS_2_ act as a ‘mediator’ for electron transfer’ from Fe^0^ to contaminants (Fig. 4) when the whole system is mechanically stirred at 400 rpm? Such a high stirring speed was explicitly selected to ensure that both Fe^0^ and FeS_2_ could be uniformly dispersed in the reaction solution^22^. This example clearly shows that using FeS_2_ to enhance the efficiency of Fe^0^/H_2_O systems is a simple tool to design more sustainable Fe^0^-based systems. However, current rationalization efforts are not really based on scientific principles^22,33^. Thus, only when the scientific principles are well-understood can better systems be designed^8,27,70^. A typical design problem is how to cope with the increased Fe^0^ dissolution specifically in column operations intrinsically prone to clogging^79,80^. Thus, in solving the enigma of the Fe^0^/FeS_2_/H_2_O system, this work leads to several avenues for sustaining the efficiency of conventional Fe^0^/H_2_O remediation systems. This result is especially important as Fe^0^-based (filtration) systems are an excellent candidate to help the international community to solve the long-lasting issue of universal safe drinking water^8,14,15,27,29,30,81,82^.

A further argument against the electrochemical nature of FeS_2_ in mediating electron transfer from Fe^0^ to contaminants (Fig. 4) is given by recent investigations in efforts to suppress FeS_2_ oxidation under environmental conditions^83–86^. For example, Seng et al.^84^ reported that Fe^0^ is able to stop FeS_2_ oxidation, and thus remediate acid mine drainage. In essence, Seng et al.^84^ investigated a contaminant-free Fe^0^/FeS_2_/H_2_O system (Table 6) and concluded that from the intrinsic properties the addition of Fe^0^ selectively suppress pyrite oxidation. Table 6 shows that the experimental conditions of Seng et al.^84^ are very close to those of remediation Fe^0^/FeS_2_/H_2_O systems. The only two distinct differences are: (i) the higher FeS_2_ mass loading (FeS_2_: Fe^0^ = 10), and (ii) the longer experimental duration (41 days versus < 10 h). By using an even longer experimental duration (41 days) and quiescent conditions (0 rpm), the present work has demonstrated the essential virtue of working under near-field conditions. In other words, it is fair to state that the Fe^0^/FeS_2_ literature is full of possibly reproducible results, but with low practical value. As already shown in Table 5, the variability of the operational conditions is a major issue and the significance of results of solid phase characterization is questionable. In fact, as seen in Table 6, a myriad of characterization tools were used to “confirm” the reducing properties of Fe^0^ for dyes^33^. In such studies species like methylene blue^70^ used herein as a ‘tracer’ of reactivity or arsenic^22^, and proven to be non-reducible in Fe^0^/H_2_O systems are quantitatively removed. The first merit of the MB method is to uncover these controversial views without solid phase analysis.

**Table 6:** Experimental conditions of selected studies using the Fe^0^/FeS_2_/H_2_O system.

Contaminant		Fe^0^		FeS_2_	Mixing rate	Duration	pH_0_	Fe^0^ characterization tools	References
X	[X] (mg L^−1^)	Type	Loading (g L^−1^)	Loading (g L^−1^)	(rpm)	(min or d)	(−)		
NB^(1)^	25.0	Powder	0.5	0.5 to 3.0	Shaken, 200	300 min	6.0	XRD^(4)^, XPS^(5)^, SEM–EDS^(6)^, XAS^(7)^ and Mössbauer spectroscopy,	^41^
TCE^(2)^	1.0	Powder	0.0 to 10.0	0.0 to 10.0	Stirred, 100	n.s. ^(3)^	n.s. ^(3)^	None	^86^
Cr(VI)	20.0	Powder	5.0	10.0	Shaken, 200	120 min	4.0	BET-N_2_ adsorption, XPS^(5)^ and SEM–EDS^(6)^	^40^
As(III)	2.0	Powder	≤ 1.0	≤ 1.0	Stirred, 400	180 min	6.8	SEM^(8)^, XPS^(5)^ and XRD^(4)^	^22^
Dyes	6.2 to 17.5	Powder	0.25 or 0.5	0.25 to 2.0	Shaken, 200	≤ 240 min	7.0	SEM–EDS^(6)^ and XPS^(5)^	^33^
Dyes	10.0	Scrap iron	5.0	2.5 to 30	Quiescent	41 d	6.5–7.0	None	TR
None	–	Powder	10.0	100.0	Shaken, 120	21 d	~ 5.6	SEM–EDX^(6)^ and ATR/FTIR^(9)^	^84^

X stands for the tested contaminant and [X] its initial concentration. It is seen that only this study used quiescent systems and the longest experimental duration. This study has also performed no solid phase Fe^0^ analysis.

^(1)^ Nitrobenzene; ^(2)^ Carbon tetrachloride; ^(3)^ n.s. = not specified; ^(4)^ XRD = X-ray diffraction; ^(5)^ XPS = X-ray photoelectron spectroscopy; ^(6)^ SEM–EDS = scanning electron microscopy—energy dispersive X-ray spectroscopy; ^(7)^ XAS = X-ray absorption spectroscopy; ^(8)^ SEM = scanning electron microscopy; ^(9)^ ATR/FTIR = attenuated total reflection /Fourier transform infrared spectroscopy; TR = This research.

The conclusion of Seng et al.^84^ supports the view presented herein that the relative kinetics of Fe^0^ and FeS_2_ oxidation determinate the preponderance of processes in Fe^0^/FeS_2_/H_2_O systems^70^. However, the reported selectivity of the process is questionable as sand and other natural minerals are also covered with FeCPs under similar conditions^29,87–89^. As an example, Song et al.^87^ reported on increased Cr^VI^ reduction in Fe^0^/sand/H_2_O systems compared to Fe^0^/H_2_O ones. The extent of coating of each aggregate (e.g. gravel, peat, pyrite, sand) depends on both the intrinsic reactivity of used Fe^0^ and the relative proportion of available materials. In the light of the kinetic arguments given herein, a re-evaluation of published works is possible, for example, the data of Sheba et al.^86^ discussing the extent of degradation of chlorinated organic compounds (RCl) by Fe^0^/FeS_2_/H_2_O systems and reporting on differential mechanisms at different Fe^0^:FeS_2_ ratios. The discussion given herein clearly suggests that if there are differential removal mechanisms, it is due to the differential extent of pH shift. Future research should be designed based on the chemistry of the systems^89^.

## Concluding remarks

The concept that adsorption and co-precipitation are the fundamental mechanisms of contaminant removal in Fe^0^/H_2_O systems is consistent with many experimental observations. In particular, quantitative dye discoloration was only observed for pH values corresponding to iron precipitation (hydroxide formation) (pH > 4.5, Fig. 3b), while selective dye discoloration promoted adsorptive removal. Further, while the role of the redox-mediated reactions in the discoloration of both dyes can only be speculatively discussed based on one’s results, it is established that the role of FeS_2_ is as follows: (i) shifting the pH to more acidic values, and (ii) enhancing contaminant removal by adsorption and co-precipitation during the subsequent pH increase by virtue of iron corrosion. Finally, negatively charged methyl orange (MO) showed no significant increase in discoloration relative to positively charged methylene blue (MB). Both MB and MO have a similar molecular size. This observation is consistent with the role of Fe^0^ as a generator of contaminant scavengers, and not as a reducing agent. This observation could explain why various As (As^III^ and As^V^)^90^ or Se (Se^IV^ and Se^VI^)^91^ species are quantitatively removed in Fe^0^/H_2_O systems, but not by aged iron oxides. Further research is needed to investigate the phenomena highlighted in the current study using a wide range of contaminants commonly occurring in drinking water and wastewaters.
